# Beef Production and Greenhouse Gas Emissions

**DOI:** 10.1289/ehp.11716

**Published:** 2008-09

**Authors:** Alex Avery, Dennis Avery

**Affiliations:** Hudson Institute, Center for Global Food Issues, Churchville, Virginia, E-mail: aavery@hughes.net

In their article discussing the impacts of farm animal production on climate change, Koneswaran and Nierenberg (2008) called for “immediate and far-reaching changes in current animal agriculture practices” to mitigate greenhouse gas (GHG) emissions. One of their recommendations was to switch to organic livestock production, stating that

Raising cattle for beef organically on grass, in contrast to fattening confined cattle on concentrated feed, may emit 40% less GHGs and consume 85% less energy than conventionally produced beef.

These claims are terribly misleading. Koneswaran and Nierenberg (2008) compared organic beef produced in Sweden (22.3 kg of carbon dioxide-equivalent GHG emissions per kilogram of beef) with unusual and resource-intensive Kobe beef production in Japan (36.4 kg of CO_2_-equivalent GHG emissions per kilogram) (Cederberg and Stadig 2003; Ogino et al. 2007).

To achieve the ultra-high fat levels in meat preferred by Japanese consumers, Japan’s wagyu cattle are raised and fattened for more than twice as long as typical U.S. beef cattle (Cattle Marketing Information Service Inc. 2007; Ogino et al. 2007). Moreover, all of the feed and forage for the Japanese animals (from birth through slaughter) must be shipped especially long distances—> 18,000 miles in the example cited. Hence, this beef has ultra-high GHG emissions and energy requirements.

According to several analyses, typical nonorganic beef production in the United States results in only 22 kg of CO_2_-equivalent GHG emissions per kilogram of beef, which is 0.3 kg less than the Swedish organic beef system (Johnson et al. 2003; Subak 1999). These comprehensive life cycle analyses, which examined all aspects of beef production and all GHG emissions, seem to definitively rule out significant reductions in GHG emissions by switching to organic beef production.

In fact, if nitrous oxide and other emissions from land conversion are included in the analysis, a large-scale shift to organic, grass-based extensive livestock production methods would increase overall GHG emissions by nearly 60% per pound of beef produced.

According to Searchinger et al. (2008), each acre of cleared land results in 10,400 lb/acre/year of CO_2_-equivalent GHG (over a 30-year period, based on estimated emissions from a proportion of each land type converted to cultivation in the 1990s). Our own analysis (Avery and Avery 2007) using conservative beef production parameters from Iowa State University’s Leopold Center for Sustainable Agriculture shows that grain-finishing cattle is at least three times more land efficient per pound of finished beef compared to grass-finishing.

Cattle industry statistics [U.S. Department of Agriculture (USDA) 2008] show that, in 2007, the United States used 2 billion bushels of corn to produce 22.16 billion lb finished grain-fed beef (17.3 million head steers and 10.2 million head heifers at average dressed weights of 830.2 and 764.8 lb, respectively). At 150 bushels/acre corn, this means we used 13.3 million acres to produce the feed grains. Converting all beef production to grass-based finishing would require at least an additional 26.6 million acres of pasture/grass to produce 2007 U.S. beef output.

Using the 22 lb of CO_2_-equivalent GHG per pound of grain-fed beef from Johnson et al. (2003) and the 22.3 lb CO_2_-equivalent GHG per pound of beef for organic grass of Cederberg and Stadig (2003), each system producing 22.16 billion lb of beef would directly and indirectly result in 487.5 and 494.2 billion lb of CO_2_-equivalent GHG emissions, respectively.

However, adding the “carbon debt” resulting from the additional cleared land required by the two-thirds less efficient grass finishing process (26.6 million acres × 10,400 lb/acre/year, or 276.6 billion lb/year) results in the organic system totaling 770 billion lb of CO_2_-equivalent GHG emissions; or 58% higher than the conventional system’s total of 487.5 billion lb.
